# Association of sexual relationship power with PrEP persistence and other sexual health outcomes among adolescent and young women in Kenya and South Africa

**DOI:** 10.3389/frph.2023.1073103

**Published:** 2023-05-30

**Authors:** Elzette Rousseau, Linxuan Wu, Renee Heffron, Jared M. Baeten, Connie L. Celum, Danielle Travill, Sinead Delany-Moretlwe, Linda-Gail Bekker, Elizabeth Bukusi, Victor Omollo, Ariane van der Straten, Gabrielle O’Malley, Jessica E. Haberer, Jennifer F. Morton, Rachel E. Johnson, Sarah T. Roberts

**Affiliations:** ^1^ Desmond Tutu HIV Centre, University of Cape Town, Cape Town, South Africa; ^2^ Departments of Global Health, Medicine and Epidemiology, University of Washington, Seattle, WA, United States; ^3^ Gilead Sciences, Inc., Seattle, WA, United States; ^4^ Wits RHI, University of the Witwatersrand, Johannesburg, South Africa; ^5^ Kenya Medical Research Institute, Kisumu, Kenya; ^6^ Department of Medicine, Centre for AIDS Prevention Studies, University of California, San Francisco, San Francisco, CA, United States; ^7^ Astra Consulting, Kensington, CA, United States; ^8^ Harvard Medical School, Harvard University, Boston, MA, United States; ^9^ Centre for Global Health, Massachusetts General Hospital, Boston, MA, United States; ^10^ RTI International, Women’s Global Health Imperative (WGHI), Berkeley, CA, United States

**Keywords:** pre-exposure prophylaxis (PrEP), PrEP persistence, sexual relationship power, sexual and reproductive health outcomes, adolescent girls and young women (AGYW)

## Abstract

**Introduction:**

Gendered power inequalities impact adolescent girls’ and young women's (AGYW) sexual and reproductive health (SRH) outcomes. We investigated the influence of sexual relationship power on AGYW's SRH outcomes, including HIV pre-exposure prophylaxis (PrEP) persistence.

**Methods:**

The POWER study in Kisumu, Kenya, and Cape Town and Johannesburg, South Africa provided PrEP to 2,550 AGYW (aged 16–25). AGYW's perceived power in their primary sexual relationship was measured among the first 596 participants enrolled using the Sexual Relationship Power Scale's (SRPS) relationship control sub-scale. Multivariable regression was used to test for (1) key sociodemographic and relationship characteristics associated with relationship power; and (2) the association of relationship power with SRH outcomes including PrEP persistence.

**Results:**

In this cohort, the mean SRPS score was 2.56 (0.49), 542 (90.9%) initiated PrEP; 192 (35.4%) persisted with PrEP at 1 month of which 46 (24.0% of 192) persisted at 6 months. SRPS were significantly lower among AGYW who cohabited with their sex partner (−0.14, 95% CI: −0.24 to −0.04, *p* = 0.01), or had ≥1 sex partner (−0.10, 95% CI: −0.19 to −0.00, *p* = 0.05). AGYW with lower SRPS were more likely to not know their partner's HIV status (aOR 2.05, 95% CI: 1.27 to 3.33, *p* < 0.01), but SRPS was not associated with PrEP persistence, STI infection, condom, or hormonal contraception use.

**Discussion:**

AGYW's reasons for initiating PrEP and reasons for continuously using PrEP may be different. While low relationship power was associated with perceived HIV vulnerability, AGYW's PrEP persistence may be influenced by more than relationship power.

## Introduction

Gendered power inequalities impact young women's sexual and reproductive health (SRH) behaviours and their access to and use of preventative health interventions (1, 2). Within intimate relationships, these power inequalities transpire through a male partner's controlling behaviours over decisions regarding safe sex, timing of sex, and sexual consent (3). In previous studies of African adolescent girls and young women (AGYW), low sexual relationship power has been associated with inconsistent condom use, lower contraceptive use, higher rates of pregnancy, physical and sexual violence, and acquisition of HIV and other sexually transmitted infections (STI) (2–9).

In sub-Sahara Africa (SSA), AGYW are disproportionately affected by sexual coercion, reproductive interference, and HIV, with this population experiencing an estimated 1,000 new HIV infections daily (10, 11). Relationship-level factors not only contribute to AGYW's susceptibility to HIV but also hinder them from adopting HIV prevention methods that need a high level of agency or influence within one's relationship (12–14). Recent HIV prevention research has focused on the development of discreet, female-controlled methods. Oral PrEP (pre-exposure prophylaxis), when taken daily, provides highly effective HIV prevention without dependence on a sexual partner (15). However, narratives from SRH and PrEP demonstration projects indicate that even though PrEP is user-controlled, AGYW often desire to disclose use and value their sexual partners’ approval of PrEP (16–18). This influences PrEP uptake (19, 20), while the fear of intimate partner violence (IPV) negatively influences PrEP persistence (continued daily adherence) (21–23). A few studies in SSA have explored the influence of relationship power on SRH outcomes with mixed evidence (3). The impact of relationship power on AGYW's PrEP persistence has not been evaluated in previous research and understanding the influence of relationship power may be important for developing strategies to support PrEP persistence in AGYW with continued HIV risk.

The POWER (Prevention Options for Women Evaluation Research) study in Kenya and South Africa offered PrEP to AGYW as part of integrated SRH services and evaluated PrEP uptake and persistence (24). In this manuscript, we describe perceived sexual relationship power in the POWER study AGYW cohort and the key sociodemographic and relationship characteristics associated with relationship power. Secondly, we investigate the influence of relationship power on AGYW's PrEP persistence and other sexual health outcomes, including contraception use, condom use, knowledge of partner HIV status, and the presence of a curable STI.

## Methods

### Research setting and study participants

Between 2017 and 2019, 2,550 HIV-uninfected AGYW (16–25 years) enrolled in the POWER study across four sites—two family planning clinics in Kisumu, Kenya; an adolescent-friendly clinic in Johannesburg (ages 18–25 only), and a mobile clinic in Cape Town, South Africa. Detailed study procedures have been described (24). Eligible participants were HIV-negative, had a primary sex partner, and reported vaginal sex in the past 3 months. Follow-up occurred 1 month after PrEP initiation and then quarterly thereafter for up to 36 months.

### Measurements

Demographic data on age, relationship status, partner cohabitation, number of sex partners, and number of children were assessed cross-sectionally at the enrollment visit. HIV vulnerability and SRH outcomes were assessed including participants’ self-reported knowledge of partner HIV status, inconsistent condom use, hormonal contraceptive use, and presence of STI. Presence of a curable STI infection was defined as a positive GeneXpert urine nucleic acid amplification test result for *Chlamydia trachomatis* (CT) and/or *Neisseria gonorrhea* (NG). Hormonal contraceptive (oral, injectable, or implant) use was categorized as either already using, wanting to start hormonal contraception, or neither on contraception nor wanting to start. Inconsistent condom use was defined as women self-reporting that they used condoms sometimes or never (versus always) in the previous 3 months. PrEP persistence was assessed among women who initiated PrEP, with non-persistence defined in the same manner as in the POWER primary analyses: ≥15 days gap in PrEP availability for daily dosing as per pharmacy records (24).

AGYW's perceived power in their primary sexual relationship was measured with the sexual relationship power scale (SRPS), widely used in HIV and reproductive health research (1, 25). The SRPS draws from the Theory of Gender and Power and the Social Exchange Theory which defines power as the amount of control one person has over decision-making in the relationship and the amount of resistance in one partner that can potentially be overcome by the other (26). The 15-item SRPS relationship control sub-scale was administered at enrollment to a convenience sample of the first approximately 150 participants at each site (*n* = 600) as part of an interviewer-administered survey. The subscale measured constructs of relationship control including physical violence, safe sex negotiation, relationship satisfaction, and relationship decision-making power on a 4-point Likert scale (1 = strongly agree to 4 = strongly disagree), with higher scores indicating more equal relationships (26). Participants with more than one sex partner were asked to respond based on their relationship with their primary partner.

### Analysis

Descriptive statistics were generated for women's demographics, behavioural characteristics, PrEP initiation, and persistence (at months one and six follow-up visits), and other SRH outcomes. The mean SRPS score for each young woman was calculated (possible range 1–4) and categorized by splitting the scale into tertiles that we labeled as lower, middle, and higher relationship power, following practices in the original paper and subsequent applications (25–28). Linear regression models were used to assess the association between each background characteristic and continuous SRPS scores at baseline. A multivariable model included age *a priori* plus all variables significant at the *p* < 0.1 level in the bivariate analyses except for marital status, which was collinear with cohabitation. All subsequent analyses used an alpha of 0.05 to assess statistical significance.

To assess the association between SRPS score tertiles and PrEP and SRH outcomes, we used logistic regression models for binary outcomes and multinomial logistic regression models for the 3-level hormonal contraceptive use outcome. Due to low retention rates during follow-up and subsequent missing data for questionnaires and lab tests (24), analysis of SRH outcomes was conducted cross-sectionally using baseline data. The analysis of PrEP persistence was conducted prospectively with missed follow-up visits inherently indicating missed refills. Multivariable models adjusted for site and age *a priori* plus partner cohabitation based on the results of the analysis of sexual relationship power predictors. Odds ratios (OR) are presented with 95% confidence intervals (95% CI). De-identified data was captured in DFcollect (DF/Net Research Inc.) and imported into SAS v.9.4 (SAS Institute Inc.) for cleaning. R version 1.4.2 was used for all analyses. Internal consistency of the SRPS was calculated at 0.85 using Cronbach's alpha.

### Ethics statement

The research was approved by the human research ethics committees of the University of Washington, University of Cape Town, Kenya Medical Research Institute, and the University of Witwatersrand. All participants provided written informed consent. Parental consent was waived for 16- and 17-year-old participants in Cape Town and Kenya, while participation at the Johannesburg site was limited to adults ages 18–25.

## Results

### Participant demographic and descriptive outcome characteristics

A total of 599 of AGYW aged 16–25 years completed the SRPS questionnaire at enrollment and 596 were included in this analysis (3 were excluded as they did not have a primary sex partner). Table 1 presents participant baseline characteristics across the three implementation sites. The participants had a median age of 21 years, 78.7% were single with a partner, 20.5% were living with their partner, and 41.1% had a child.

**Table 1:** Participant background characteristics and descriptive SRH outcomes.

	Overall	Cape Town	Johannesburg	Kisumu
Total number of participants	596	146 (24.5%)	150 (25.2%)	300 (50.3%)
**Background characteristics**				
Age (continuous), median (IQR)	21 (19–22)	20 (18–22)	21 (19–22)	21 (19–23)
	*n* (%)	*n* (%)	*n* (%)	*n* (%)
**Age (categorical)**				
16–17 years	48 (8.1%)	29 (19.9%)	0 (0.0%)^a^	19 (6.3%)
18–21 years	315 (52.8%)	70 (47.9%)	95 (63.3%)	150 (50.0%)
22–25 years	233 (39.1%)	47 (32.2%)	55 (36.7%)	131 (43.7%)
**Marital status**				
Single, no partner	14 (2.3%)	5 (3.4%)	5 (3.3%)	4 (1.3%)
Single, with partner	469 (78.7%)	139 (95.2%)	141 (94.0%)	189 (63.0%)
Married, husband has one wife	103 (17.3%)	2 (1.4%)	4 (2.7%)	97 (32.3%)
Other	10 (1.7%)	0 (0.0%)	0 (0.0%)	10 (3.3%)
**Lives with sexual partner**				
No	470 (79.4%)	141 (97.9%)	137 (91.3%)	192 (64.4%)
Yes	122 (20.6%)	3 (2.1%)	13 (8.7%)	106 (35.6%)
**>1 sex partner**				
No	491 (82.8%)	131 (89.7%)	137 (91.9%)	223 (74.8%)
Yes	102 (17.2%)	15 (10.3%)	12 (8.1%)	75 (25.2%)
**Number of living children**				
0	349 (58.7%)	109 (75.7%)	100 (66.7%)	140 (46.7%)
≥1	245 (41.3%)	35 (24.3%)	50 (33.3%)	160 (53.3%)
**SRH outcomes at enrollment**				
**Partner HIV status**				
Unknown HIV status	397 (66.7%)	111 (76.0%)	77 (51.3%)	209 (70.0%)
Known, partner HIV positive	27 (4.6%)	8 (5.5%)	4 (2.7%)	15 (5.0%)
Known, partner HIV negative	171 (28.7%)	27 (18.5%)	69 (46.0%)	75 (25.0%)
**Inconsistent condom use**				
No (always use condoms)	83 (14.0%)	29 (19.9%)	25 (16.7%)	29 (9.8%)
Yes (sometimes/never use condoms)	509 (86.0%)	125 (80.1%)	125 (83.3%)	267 (90.2%)
**STI (GC/NG) infection**				
Negative	364 (69.3%)	72 (62.6%)	73 (64.6%)	219 (73.2%)
Positive	163 (30.7%)	43 (37.4%)	40 (35.4%)	80 (26.8%)
**Hormonal contraceptive**				
On contraceptive	221 (37.8%)	65 (45.1%)	60 (42.3%)	96 (32.1%)
Wants contraceptive	135 (23.1%)	57 (39.6%)	44 (31.0%)	34 (11.4%)
Neither	229 (39.1%)	22 (15.3%)	38 (26.7%)	169 (56.5%)
**SRH outcomes assessed during follow-up**				
**PrEP initiated ever**				
No	54 (9.1%)	3 (2.1%)	5 (3.3%)	46 (15.3%)
Yes	542 (90.9%)	143 (97.9%)	145 (96.7%)	254 (84.7%)
**PrEP persistent through 1 month**				
No	350 (64.6%)	102 (71.3%)	84 (57.9%)	164 (64.6%)
Yes	192 (35.4%)	41 (28.7%)	61 (42.1%)	90 (35.4%)
**PrEP persistent through 6 months^b^**				
No	146 (76.0%)	37 (90.2%)	31 (50.8%)	78 (86.7%)
Yes	46 (24.0%)	4 (9.8%)	30 (49.2%)	12 (13.3%)

^a^ Johannesburg only enrolled AGYW between the ages of 18–25.

^b^ PrEP persistence at 6 months was calculated including only participants who persisted through 1 month of PrEP use.

At enrollment, 66.7% of participants did not know their partner's HIV status, 86.0% reported inconsistent condom use for the preceding 3 months, and 30.7% had an STI (GC/NG). Over one third (37.8%) were on hormonal contraceptives and 23.1% wanted to start at that visit, while 39.1% were not interested in being on contraception. PrEP was initiated by 542 (90.9%) of AGYW at some point during their study participation, 192 (35.4% of 542) received a refill at 1 month, and 46 (24.0% of 192) persisted and obtained PrEP refills through 6 months of follow-up. The characteristics of participants in this analysis sample were similar to the overall POWER study cohort (*N* = 2,550).

### Participants’ sexual relationship power

This cohort had a mean SRPS score of 2.56 (0.49). SRPS score tertile ranges were 1.06–2.38 for the lower third, 2.38–2.75 for the middle third, and 2.75–3.69 for the higher third. Responses to individual questions indicated that male partners had a substantial level of control in the relationships (Table 2): 60.4% agreed that their partners might be having sex with someone else, 33.6% believed that their partner had more say about important decisions, 28.9% indicated that their partner would get angry and 23.2% violent if asked to use a condom, 67.5% say that her partner always wants to know where she is, 35% tell her whom she can spend time with, 38.6% say that in disagreements their partner gets his way most times, and 18.6% reported that they felt trapped or stuck in their relationships. The mean score was significantly lower among participants from Kisumu (mean = 2.35) than from Cape Town (mean = 2.73) or Johannesburg (mean = 2.81, *p* < 0.01).

**Table 2:** Sexual relationship power: percentage of women who agree/strongly agree with individual items in the SRPS and the mean scale scores.

	Total	Cape Town	Johannesburg	Kisumu	*p*
Total number of participants	596	146	150	300	
*SRPS item = Strongly agree/Agree n (%)*	*n (%)*	*n (%)*	*n (%)*	*n (%)*	
If I asked my partner to use a condom, he would get violent.	138 (23.2%)	12 (8.2%)	20 (13.3%)	106 (35.3%)	<0.01
If I asked my partner to use a condom, he would get angry.	172 (28.9%)	24 (16.4%)	27 (18.0%)	121 (40.3%)	<0.01
Most of the time, we do what my partner wants to do.	222 (37.2%)	31 (21.2%)	39 (26.0%)	152 (50.8%)	<0.01
My partner will not let me wear certain things.	225 (37.8%)	50 (34.5%)	45 (30.0%)	130 (43.5%)	0.01
When my partner and I are together, I am pretty quiet.	124 (20.8%)	32 (21.9%)	19 (12.7%)	73 (24.3%)	0.01
My partner has more say than I do about important decisions that affect us.	200 (33.6%)	28 (19.3%)	31 (20.7%)	141 (47.3%)	<0.01
My partner tells me who I can spend time with.	210 (35.2%)	32 (22.1%)	33 (22.1%)	145 (48.5%)	<0.01
If I asked my partner to use a condom, he would think I am having sex with other people.	218 (36.6%)	50 (34.2%)	34 (22.7%)	134 (44.7%)	<0.01
I feel trapped or stuck in our relationship.	111 (18.6%)	17 (11.7%)	23 (15.3%)	71 (23.7%)	<0.01
My partner does what he wants, even if I do not want him to.	206 (34.6%)	28 (19.3%)	44 (29.3%)	134 (44.7%)	<0.01
I am more committed to our relationship than my partner is	179 (30.0%)	46 (31.7%)	56 (37.3%)	77 (25.7%)	0.03
When my partner and I disagree, he gets his way most of the time.	230 (38.6%)	42 (29.0%)	39 (26.2%)	149 (49.7%)	<0.01
My partner gets more out of our relationship than I do.	138 (23.2%)	30 (20.7%)	29 (19.3%)	79 (26.3%)	0.18
My partner always wants to know where I am.	402 (67.4%)	96 (66.2%)	84 (56.0%)	222 (74.0%)	<0.01
My partner might be having sex with someone else.	360 (60.4%)	92 (63.4%)	65 (43.3%)	203 (67.7%)	<0.01
*SRPS Score [mean (SD)]*	*2.56* *(0.49)*	*2.73* *(0.41)*	*2.81* *(0.48)*	*2.35* *(0.44)*	*<0* *.* *01*

### Demographic factors associated with sexual relationship power

In the univariable analysis, demographic factors significantly associated with lower sexual relationship power among AGYW included age, being from Kisumu, living with her sex partner, having children, and having more than one sex partner (Table 3). Study site (aOR −0.31, 95% CI: −0.40 to 0.21, *p* < 0.01) and partner cohabitation (aOR −0.14, 95% CI: −0.24 to −0.04, *p* < 0.01) remained significantly associated with lower relationship power in the multivariable analysis.

**Table 3:** Demographic factors associated with sexual relationship power: univariable and multivariable analysis.

Factors	Mean SRPS (SD)	Univariable regression		Multivariable regression	
		Coefficient (95% CI)	*p*-value	Coefficient (95% CI)	*p*-value
Age, continuous	2.56 (0.49)	−0.02 (−0.03 to 0.01)	0.07	0.01 (−0.01 to 0.02)	0.69
**Study site**					
Cape Town	2.73 (0.41)				
Johannesburg	2.81 (0.48)	0.09 (−0.01 to 0.19)	0.09	0.08 (−0.02 to 0.18)	0.11
Kisumu	2.35 (0.44)	−0.38 (−0.46 to −0.29)	<0.01	−0.31 (−0.40 to 0.21)	<0.01
**Marital status^a^**					
Single, no partner	2.61 (0.52)				
Single, with partner	2.64 (0.46)	0.03 (−0.21 to 0.28)	0.80		
Married, husband has one wife	2.25 (0.45)	−0.35 (−0.61 to −0.09)	0.01		
Other	1.93 (0.38)	−0.68 (−1.06 to −0.31)	<0.01		
**Lives with sexual partner**					
No	2.63 (0.47)				
Yes	2.31 (0.48)	−0.32 (0.42 to −0.23)	<0.01	−0.14 (−0.24 to −0.04)	0.01
**Number of living children**					
0	2.65 (0.48)				
≥1	2.44 (0.49)	−0.21 (−0.28 to −0.13)	<0.01	−0.07 (−0.15 to 0.01)	0.07
**>1 sex partner**					
No	2.59 (0.50)				
Yes	2.40 (0.42)	−0.19 (−0.29 to −0.09)	0.05	−0.10 (−0.19 to 0.00)	0.05

^a^ Omitted from multivariable analysis due to collinearity with cohabitation.

### Relationship power and sexual reproductive health outcomes

Baseline sexual relationship power was not significantly associated with PrEP persistence at 1 (OR 0.72, 95% CI: 0.41 to 1.11, *p* = 0.13) or 6 months (OR 0.79, 95% CI: 0.35 to 1.71, *p* = 0.55) of follow-up (Table 4). In the cross-sectional analyses of baseline SRH outcomes, women with lower relationship power had twice the odds of not knowing a partner's HIV status than women with higher relationship power (aOR 2.05, 95% CI: 1.27 to 3.33, *p* < 0.01). Additionally, women with lower relationship power were significantly more likely to report inconsistent condom use during the past 3 months (OR 1.95, 95% CI: 1.07 to 3.67, *p* = 0.03) in the univariable model, but not in the multivariable model (aOR 1.29, 95% CI: 0.67 to 2.56, *p* = 0.45). Relationship power was not associated with the presence of an STI (GC/NG) (aOR 0.93, 95% CI: 0.56 to 1.33, *p* = 0.77). Compared to women with higher relationship power, women with lower relationship power were less likely to be on hormonal contraception (OR 0.45, 95% CI: 0.29 to 0.72, *p* < 0.01) or wanting to start hormonal contraception (OR 0.42, 95% CI: 0.25 to 0.72, *p* < 0.01), although this was not significant in the multivariable analysis (aOR 0.65, 95% CI: 0.39 to 1.09, *p* = 0.11).

**Table 4:** Associations between baseline sexual relationship power and prEP and SRH outcomes: univariable and multivariable analyses.

Outcomes	*n*/*N*	%	Univariable regression		Multivariable regression^a^	
			OR (95% CI)	*p*-value	OR (95% CI)	*p*-value
**Prospective outcomes**						
**PrEP persistence through 1 month (*N* = 542)**						
Higher SRPS	74/184	40.2%	Ref		Ref	
Middle SRPS	62/186	33.3%	0.74 (0.49 to 1.14)	0.17	0.78 (0.50 to 1.22)	0.28
Lower SRPS	56/172	32.6%	0.72 (0.46 to 1.11)	0.13	0.69 (0.42 to 1.11)	0.12
**PrEP persistence through 6 months (*N* = 192)**						
Higher SRPS	22/74	29.7%	Ref		Ref	
Middle SRPS	10/62	16.1%	0.45 (0.19 to 1.03)	0.07	0.8 (0.30 to 2.05)	0.64
Lower SRPS	14/56	25%	0.79 (0.35 to 1.71)	0.55	1.44 (0.56 to 3.77)	0.45
**Cross-sectional outcomes**						
**Unknown partner HIV status (*N* = 595)**						
Higher SRPS	115/199	57.8%	Ref		Ref	
Middle SRPS	136/198	68.7%	1.6 (1.06 to 2.42)	0.02	1.32 (0.85 to 2.05)	0.22
Lower SRPS	146/198	73.7%	2.05 (1.35 to 3.14)	<0.01	2.05 (1.27 to 3.33)	<0.01
**Inconsistent condom use (*N* = 592)**						
Higher SRPS	166/199	83.4%	Ref		Ref	
Middle SRPS	166/198	83.8%	1.03 (0.61 to 1.76)	0.91	0.91 (0.51 to 1.59)	0.73
Lower SRPS	177/195	90.8%	1.95 (1.07 to 3.67)	0.03	1.29 (0.67 to 2.56)	0.45
**STI (GC/NG) infection (*N* = 527)**						
Higher SRPS	58/172	33.7%	Ref		Ref	
Middle SRPS	54/168	32.1%	0.93 (0.59 to 1.46)	0.76	1 (0.62 to 1.61)	0.99
Lower SRPS	51/187	27.3%	0.74 (0.47 to 1.16)	0.18	0.93 (0.56 to 1.53)	0.77
**Contraceptive Use or Interest^b^ (*N* = 585)**						
**On contraceptive (vs. not on contraceptive) (*N* = 450)**						
Higher SRPS	83/142	58.5%	Ref		Ref	
Middle SRPS	76/149	51.0%	0.74 (0.47 to 1.18)	0.20	0.79 (0.47 to 1.30)	0.35
Lower SRPS	62/159	39.0%	0.45 (0.29 to 0.72)	<0.01	0.65 (0.39 to 1.09)	0.11
**Wants contraceptive (vs. not on contraceptive) (*N* = 364)**						
Higher SRPS	53/112	47.3%	Ref		Ref	
Middle SRPS	45/118	38.1%	0.69 (0.41 to 1.16)	0.16	0.79 (0.44 to 1.43)	0.43
Lower SRPS	37/134	27.6%	0.42 (0.25 to 0.72)	<0.01	0.9 (0.48 to 1.68)	0.74

^a^ Site, age, and lives with sexual partner were adjusted in multivariable regression.

^b^ Contraceptive results based on multinomial model: on contraception or wanting contraception vs. not on contraception.

## Discussion

In this PrEP implementation study the cohort consisted primarily of young women who were single with a partner and showed high oral PrEP initiation, but low persistence. AGYW demonstrated high HIV vulnerability through reports of having a partner of unknown HIV status, inconsistent condom use, and multiple concurrent partnerships, and through the presence of a curable STI (GC/NG) at enrollment, which is compatible with other studies in similar contexts (2, 8, 11, 29, 30). In this study, we explored the association of sexual relationship power with SRH outcomes and found that lower relationship power was associated with several factors linked to HIV vulnerability, which may have encouraged PrEP uptake, but relationship power did not predict PrEP persistence.

Intimate relationship dynamics are a known driver of PrEP uptake and may have supported PrEP interest in this cohort in which more than 90% initiated PrEP. Previous research in South Africa indicates higher PrEP interest and uptake in younger women in short-term relationships with higher-risk partners (2). In this cohort, lower relationship power was associated with study site (Kenya), sex partner cohabitation, multiple concurrent relationships, and not knowing a partner's HIV status. Prior research has found that higher commitment relationships, where partners are co-habiting and have children (observed at higher rates in the Kisumu group), are more evident of male dominance, with some or no female partner autonomy (3, 28, 31) and may pose greater risks if partners did not approve of their use of SRH services and HIV prevention methods (21, 25, 31). In addition, Kenyan women report higher rates of lifetime partner violence and adhere to more traditional and restrictive gender norms than Cape Town and Johannesburg, which lowers relationship power and reproductive health (32, 33). Furthermore, having more than one sex partner has previously been connected to lower relationship power and higher IPV (34, 35). In turn, this lower relationship power influences HIV vulnerability in that AGYW with lower relationship power are less likely to discuss or know their partner's HIV status and more likely to use condoms inconsistently with these partners (2). This cohort had a slightly lower overall mean SRPS score than similar populations in SSA and displayed higher proportions of believing that their partner had other sexual partners and that their partner will get angry or violent when asked to use a condom (3, 34). Previous research has shown that lower SRP is linked to HIV incidence, which may possibly account for the lower SRPS scores in this cohort of AGYW who decided that their vulnerability to HIV is so high that PrEP as HIV prevention was sought (3, 5, 25, 34, 35). Lower SRPS scores may be valuable in identifying AGYW with HIV vulnerability who could benefit from PrEP as an HIV prevention mechanism that is user-controlled and does not rely on a partner's permission. Understanding the role of AGYW's sexual relationship in the adoption of prevention behaviors and integrating support mechanisms for relationship power dynamics in SRH services may be beneficial in demand creation and uptake of PrEP among AGYW in need of prevention methods. In addition, HIV prevention may be further optimized for AGYW with lower relationship power by closing the HIV testing gap with their male partners.

In this cohort of African AGYW, relationship power did not predict baseline STI infection, condom use during the prior 3 months, or being on or wanting to start hormonal contraception. And sexual relationship power was not associated either positively or negatively with PrEP persistence. Recent research with AGYW PrEP users, including qualitative findings from this study, has shown that PrEP uptake and persistence early in the user journey are influenced by disclosure, social support, and PrEP stigma, all shaped to a degree by relationship dynamics and young women's need for relationship preservation (36–38). Longer-term PrEP persistence in AGYW, however, is likely influenced by more factors than only sexual relationship power. Research highlights the role of accessibility of PrEP services, healthcare provider stigma, level of trust in an intimate relationship, pill-taking fatigue and desire for long-acting PrEP, and social support from the people sharing AGYW's living space (primarily family and not a sexual partner in this cohort), which in combination may have overwhelmed any effect of sexual relationship power on persistence (12, 39–43). Supporting AGYW in effectively using PrEP will likely need a multi-faceted, yet tailored, response from providers of which intimate relationship dynamics will be a component.

This study had several limitations. Firstly, the SRPS questionnaire was only administered to AGYW interested in PrEP; therefore we could not test whether lower relationship power is a barrier to AGYW initiating PrEP, and our estimates of the level of relationship power in this population may not be generalizable to AGYW who are not interested in PrEP. Secondly, the SRPS questionnaire was interviewer-administered and social desirability might have influenced women to underreport potentially stigmatizing relationship characteristics, including IPV and control in their relationships. Thirdly, AGYW's responses to the SRPS scale were based on their primary partner at baseline, which may be different from their partner at months one and six of PrEP follow-up. Finally, the overall study did not capture information on planned PrEP pauses and continued HIV vulnerability among those who discontinued PrEP; therefore, the practice of prevention-effective adherence (only taking PrEP during periods with actual HIV vulnerability) (44) may have been misinterpreted as lack of persistence in some instances.

To our knowledge, this is the first study to examine the association of sexual relationship power and PrEP persistence among AGYW in SSA. Taken together, these results suggest that AGYW's reasons for initiating PrEP and reasons for continuously using PrEP may be different. While relationship dynamics and their role in HIV vulnerability may influence PrEP uptake, AGYW's PrEP persistence may be influenced by more than relationship power. Identifying and addressing barriers and facilitators for effective PrEP use in AGYW in SSA remains an important research objective.

## Data Availability

The raw data supporting the conclusions of this article will be made available by the authors, without undue reservation.
